# Supplementary material for: Lactic Acid Influences Iron Assimilation by a Fungal Pathogen via the Iron Reductive Uptake Pathway
Source: Microbiologyopen. 2025 Dec 16;14(6):e70167. doi: 10.1002/mbo3.70167 (PMC12706630; doi:10.1002/mbo3.70167)
Supplement: Supplementary file 1 — Figure S1: Transcriptional impact of lactate and pH in C. albicans Venn diagram showing the number of genes differentially induced at least at one time point in cells cultured in media with or without lactic acid at pH 5 and 7. [file MBO3-14-e70167-s002.pdf]

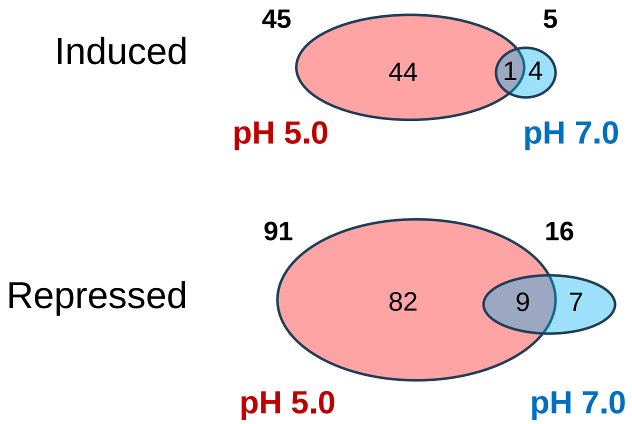

Figure S1. Venn diagram showing the number of differentially induced genes at least one time point in cells cultured in the presence or absence of lactate in the medium at pH 5.0 and 7.0.
