# Supplementary material for: Lactic Acid Influences Iron Assimilation by a Fungal Pathogen via the Iron Reductive Uptake Pathway
Source: Microbiologyopen. 2025 Dec 16;14(6):e70167. doi: 10.1002/mbo3.70167 (PMC12706630; doi:10.1002/mbo3.70167)
Supplement: Supplementary file 2 — Figure S2: Regulation of alternative iron acquisition mechanisms during lactate administration. Fold change (log2) of genes differently expressed in the presence of lactate related to iron, zinc, and copper transport (pH 5) based on the transcriptome analysis. [file MBO3-14-e70167-s001.pdf]

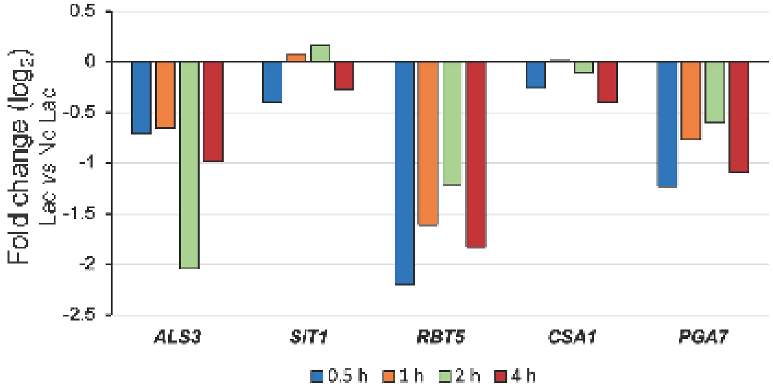

Figure S2.- Fold change (log2) of genes differently expressed in the presence of lactate related to iron, zinc, and copper transport (pH 5) based on the transcriptome analysis
